# Plasma Lipopolysaccharide-Binding Protein (LBP) Is Induced in Critically Ill Females with Gram-Negative Infections—Preliminary Study

**DOI:** 10.3390/idr17010010

**Published:** 2025-01-28

**Authors:** Alexander Utrata, Niklas Schmidtner, Patricia Mester, Stephan Schmid, Martina Müller, Vlad Pavel, Christa Buechler

**Affiliations:** Department of Internal Medicine I, Gastroenterology, Hepatology, Endocrinology, Rheumatology, and Infectious Diseases, University Hospital Regensburg, 93053 Regensburg, Germany; alexander.utrata@stud.uni-regensburg.de (A.U.); niklas.schmidtner@stud.uni-regensburg.de (N.S.); patricia.mester@klinik.uni-regensburg.de (P.M.); stephan.schmid@klinik.uni-regensburg.de (S.S.); martina.mueller-schilling@klinik.uni-regensburg.de (M.M.); vlad.pavel@klinik.uni-regensburg.de (V.P.)

**Keywords:** COVID-19, liver cirrhosis, sex, survival, lipopolysaccharide-binding protein

## Abstract

Background/Objectives: Men are more susceptible to sepsis than women, but the underlying pathways have not been fully clarified. Lipopolysaccharide-binding protein (LBP) is an acute-phase protein that is highly elevated in sepsis. Experimental evidence shows that LBP increases to a much greater extent in male than in female mice following exposure to lipopolysaccharide. However, gender-specific studies of circulating LBP levels in sepsis patients are scarce. Methods: In the plasma of 189 patients with systemic inflammatory response syndrome (SIRS), sepsis, and septic shock, LBP levels were measured by enzyme-linked immunosorbent assay. Results: Patients with liver cirrhosis had reduced circulating LBP levels, regardless of gender. Further analysis within the non-cirrhotic patients showed no significant differences in LBP levels between sexes in patients with SIRS, sepsis, and septic shock. Ventilation, dialysis, and vasopressor therapy had no effect on LBP levels in either sex. A positive correlation between LBP and C-reactive protein was observed in the total cohort, males, and females. Infection with Gram-negative or Gram-positive bacteria had no effect on plasma LBP levels in males. However, female patients with Gram-negative infection had increased plasma LBP levels compared to females with negative and Gram-positive blood cultures, and 70 µg/mL LBP discriminates Gram-negative infections in females with a sensitivity of 88% and a specificity of 74%. Infection with SARS-CoV-2 did not change plasma LBP levels in either men or women. Female patients who did not survive had lower plasma LBP levels compared to female survivors and male non-survivors. Conclusions: This investigation highlights the influence of sex on plasma LBP levels in SIRS/sepsis patients, suggesting that LBP could be a sex-specific biomarker in critically ill patients.

## 1. Introduction

Sepsis is caused by bacterial, fungal, and viral infections [1,2]. Lipopolysaccharide-binding protein (LBP) binds with the lipopolysaccharide (LPS) of Gram-negative bacteria to form LPS-LBP complexes. LBP is essential for the transfer of LPS to toll-like receptor (TLR) 4, triggering cellular responses to LPS. It has also been shown that LBP enhances the immune response to Gram-positive bacteria. LBP is now recognized as a pattern recognition receptor that transfers microbial ligands to receptors such as TLR4 [3]. Antibodies that neutralize the binding of LPS to LBP have protected mice from endotoxemic shock when injected simultaneously with LPS [4]. However, high levels of LBP, such as those found in patients with severe sepsis or septic shock, have been shown to inhibit the LPS response of human macrophages, suggesting a protective role for elevated serum LBP in sepsis [5].

In serum from healthy controls, LBP levels range from 5 to 15 µg/mL. However, the physiological role of LBP is comparatively less well understood. Mice fed a standard diet developed liver inflammation, oxidative stress, and fibrosis when LBP was knocked down in the liver [6]. It has also been reported that LBP knock-out mice are protected from toxic liver injury [7,8]. This is an indication of the opposite role of relatively low levels of LBP in physiological conditions and usually high levels of LBP in pathological states [6,7,8].

Higher levels of circulating LBP have been observed in cirrhosis of the liver and in other conditions such as coronary heart disease, diabetes, and inflammatory bowel disease [3,9]. Strongly increased serum LBP levels have been described in sepsis and septic shock [3]. Serum levels of LBP were approximately six times higher in patients with systemic inflammatory response syndrome (SIRS) and sepsis compared to healthy controls, and were further elevated in patients with septic shock. Serum LBP levels in patients with infectious and non-infectious diseases were similar and did not discriminate between survivors and non-survivors [10]. However, a second study found that levels of LBP were higher in patients with bacteremia compared to non-bacteremic patients [11]. Although the severe sepsis cohort had higher LBP levels compared to patients with less severe sepsis, LBP was not found to be related to survival [11]. In an additional cohort, there was no significant difference in LBP serum levels between severe septic patients with Gram-negative, Gram-positive, and fungal infections, and LBP levels were not associated with disease severity [12].

Severe acute respiratory syndrome coronavirus type 2 (SARS-CoV-2) infection is a newer cause of sepsis [13,14]. Patients with severe Coronavirus disease 2019 (COVID-19) had higher plasma LBP levels compared to patients with mild conditions [15]. The study found that LBP was positively associated with inflammation biomarkers, including C-reactive protein (CRP), interleukin-6, and lactate dehydrogenase, as well as with the percentage of lymphocytes and neutrophils [15]. Furthermore, systemic LPS levels in COVID-19 patients were higher compared to controls and resulted from increased microbial translocation in severe illness [16]. The spike protein of SARS-CoV-2 binds to LPS, which enables low levels of this endotoxin to activate nuclear factor kappa B [17]. Increased LBP levels in COVID-19 may therefore further aggravate disease severity.

Recent studies suggest that being male is a risk factor for severe illness and death from COVID-19 [18]. Males also have a higher incidence and severity of sepsis [19]. A different host response to pathogens of males and females has been described. When eight-week-old mice were challenged with LPS, male animals exhibited a significantly greater response, with higher levels of circulating interleukin-6 and LBP [20]. A study where 12-month-old mice were injected with LPS observed higher plasma cytokine levels and greater sickness in female mice [21]. Women at a mean age of 30 years were shown to mount stronger pro-inflammatory responses to low-dose endotoxin injections than age-matched males [22]. Moreover, exacerbated inflammatory responses to acute COVID-19 respiratory tract infection were observed in male patients, even when males and females were matched for disease severity [23]. Thus, females and males respond differently to LPS and bacterial and viral infections. Higher age may enhance the immune response of both sexes and pre- and postmenopausal women may respond differently because of the protective effects of estrogens [24,25].

Clinically, C-reactive protein (CRP) is the most commonly measured biomarker for inflammatory diseases [26]. However, patients with sepsis and underlying liver cirrhosis have reduced CRP levels due to impaired hepatic synthesis of this acute-phase protein [27]. On the other hand, patients with liver cirrhosis have higher CRP in comparison to healthy controls because liver cirrhosis may lead to a systemic state of inflammation [27]. LBP is also an acute-phase protein mostly released by hepatocytes and is elevated in the serum/plasma of patients with chronic liver diseases [9,28]. Besides liver cirrhosis, pancreatitis is a risk factor for sepsis and is also associated with increased LPS levels [29,30,31]. These patients may have symptoms of sepsis without an underlying bacterial infection [32]. Whether plasma LBP levels in critically ill patients with liver cirrhosis are similarly reduced as CRP or change in patients with underlying pancreatitis needs to be evaluated.

Despite evidence that females and males differentially respond to LPS [22], potentially due to variations in the upregulation of circulating LBP levels [20], no study has performed a sex-specific analysis of LBP in human SIRS/sepsis patients. The aim of our study was to conduct a sex- and disease-specific analysis of plasma LBP levels in critically ill patients.

## 2. Materials and Methods

### 2.1. Study Cohort

Between August 2018 and January 2024, we collected plasma samples from 189 patients at the University Hospital of Regensburg. We categorized patients according to the Sepsis-3 criteria for sepsis and septic shock [33] and the systemic inflammatory response syndrome (SIRS) criteria for SIRS [34]. The four SIRS criteria of whom two or more must apply for SIRS diagnosis are: (1) body temperature of >38 or <36 °C, (2) heart rate >90/min, (3) respiratory rate >20/min or PaCO_2_ <32 mmHg (4.3 kPa), and (4) white blood cell count >12,000/mm^3^ or <4000/mm^3^ or >10% immature bands [34,35]. Sepsis is a life-threatening organ dysfunction and is related to an acute change of the Sequential [Sepsis-related] Organ Failure Assessment (SOFA) score ≥2 points. The SOFA score assesses the neurologic, blood, liver, and kidney performance and respiration function [33]. Septic shock is a severe form of sepsis and vasopressor therapy is needed to elevate mean arterial pressure ≥65 mm Hg. Patients have lactate levels >2 mmol/L (18 mg/dL) despite adequate fluid resuscitation [33].

The patients had different causes of SIRS (46 patients), sepsis (51 patients), or septic shock (92 patients). Our cohort included patients with COVID-19. Plasma samples from the 25 COVID-19 patients were collected between October 2020 and January 2023. All COVID-19 patients included in our study were hospitalized for SARS-CoV-2 infection and all had sepsis or septic shock due to SARS-CoV-2 infection.

Patients with viral hepatitis, human immunodeficiency virus infection, or multidrug-resistant infections at admission were excluded.

Common comorbidities were neoplasms such as adenocarcinoma and colorectal cancer (13.5%), autoimmune diseases (7.7%), hematological diseases such as acute lymphoblastic leukemia and acute promyelocytic leukemia (7.7%). A total of 7.1% of patients were immunosuppressed after organ transplantation.

Laboratory values were obtained from the Institute of Clinical Chemistry and Laboratory Medicine at the University Hospital of Regensburg, and microbiological tests were performed by the Institute of Clinical Microbiology and Hygiene at our university hospital.

### 2.2. Sample Collection and Handling

We took blood samples from patients within 12 to 24 h of their admission to the intensive care unit, using EDTA as the anticoagulant (S-Monovette^®^EDTA K3E, Sarstedt, Nümbrecht, Germany). After blood collection, the sample was gently inverted three times as recommended by the supplier. At 30 min after blood collection, the samples were centrifuged at 2000 g for 15 min at room temperature. Plasma was aliquoted and stored at −80 °C until use. Samples were thawed immediately before use.

Plasma was collected between August 2018 and January 2024 and some samples were stored at −80 °C for more than 6 years. The stability of LBP in frozen plasma has not been analyzed as far as we know. Plasma LBP levels of 13 samples collected in 2018 and of 13 samples collected in 2024 did not differ (*p* = 0.095) and LBP levels may not be strongly degraded during storage.

### 2.3. LBP ELISA

We took blood samples from patients within 12 to 24 h of their admission to the intensive care unit, using EDTA as the anticoagulant. We used the human LBP ELISA Kit (Thermo Fisher Scientific; Hennigsdorf, Germany) according to the manufacturer’s instructions, with a plasma dilution of 1:1000 for analysis.

### 2.4. Statistical Analysis

We present the data using boxplots, which display the minimum and maximum LBP values, the median, and the first and third quartiles. Outliers are indicated as individual circles or asterisks. LBP levels in the plasma of patients were not normally distributed (Kolmogorov-Smirnov *p* = 0.006) and Shapiro-Wilk (*p* < 0.001). Except age all other parameters were not normally distributed and non-parametric tests were used. The tables list the median, minimum, and maximum values. We used the following statistical tests: (1) non-parametric Wilcoxon test, (2) non-parametric Kruskal-Wallis test, (3) Chi-Square test, (4) Receiver operating characteristics curve and Youden index for evaluating the biomarker effectiveness and (5) Spearman’s correlation, using IBM SPSS Statistics 26.0 software. A significance level of *p* < 0.05 was applied.

## 3. Results

### 3.1. LBP in Plasma of SIRS/Sepsis Patients

LBP was measured in the plasma of 189 patients with SIRS/sepsis and 43 controls. Patients and controls were matched for sex, but controls were younger compared to SIRS/sepsis patients (Table 1).

**Figure 1:**
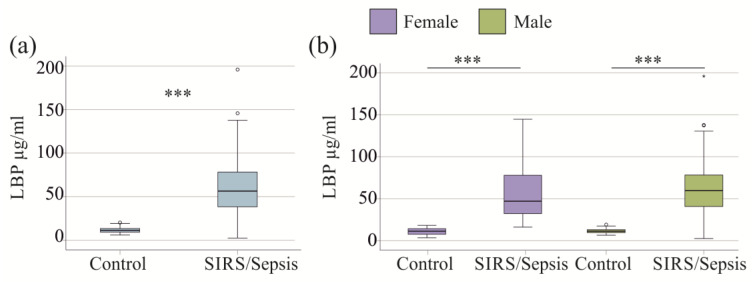
LBP in plasma of controls and SIRS/sepsis patients. (**a**) Plasma LBP levels of the 43 controls and the 189 SIRS/sepsis patients. The statistical test used: Mann–Whitney-U-test; (**b**) Plasma LBP levels of the 18 female controls, the 51 female SIRS/sepsis patients, the 25 male controls, and the 138 male SIRS/sepsis patients. Statistical test used: Kruskal-Wallis test.*** *p* < 0.001.

Among the 189 patients with SIRS or sepsis, the median plasma LBP concentration was 56.4 (2.5–196.0) µg/mL. In comparison, the 43 controls had a median concentration of 12.2 µg/mL, ranging from 5.7 to 20.3 µg/mL (*p* < 0.001; Figure 1a). Men and women had similar LBP levels in the control (*p* = 0.065) and the patient cohort (*p* = 0.408) (Figure 1b). When compared by gender, LBP levels were higher in both male and female SIRS/sepsis patients than in the respective controls (*p* < 0.001 for both; Figure 1b). These data show that the plasma levels of LBP are similarly elevated in SIRS/sepsis patients of both sexes.

In the entire patient cohort, plasma LBP negatively correlated with age (correlation coefficient r = −0.184, *p* = 0.022) but not with body mass index (BMI) (r = −0.074, *p* = 0.364). In women, the correlations of LBP levels with age (r = −0.054, *p* = 0.705) and BMI (r = −0.050, *p* = 0.728) were not significant. In male patients, no significant correlations were observed between plasma LBP levels and age (r = −0.153, *p* = 0.073) or BMI (r = −0.021, *p* = 0.812).

### 3.2. LBP in Plasma of SIRS/Sepsis Patients with and Without Liver Cirrhosis

LBP, which is abundant in hepatocytes, is increased in non-septic patients with liver cirrhosis [9]. A comparison of the total patient cohort and the subgroup of patients without liver cirrhosis revealed that C-reactive protein (CRP) was higher after the exclusion of patients with cirrhosis. The CRP of patients with cirrhosis was 67 (9–236) mg/L and that of patients without cirrhosis was 174 (4–697) mg/L (*p* < 0.001). All other parameters were similar between the entire patient cohort and the patients without liver cirrhosis (Table 1).

**Figure 2:**
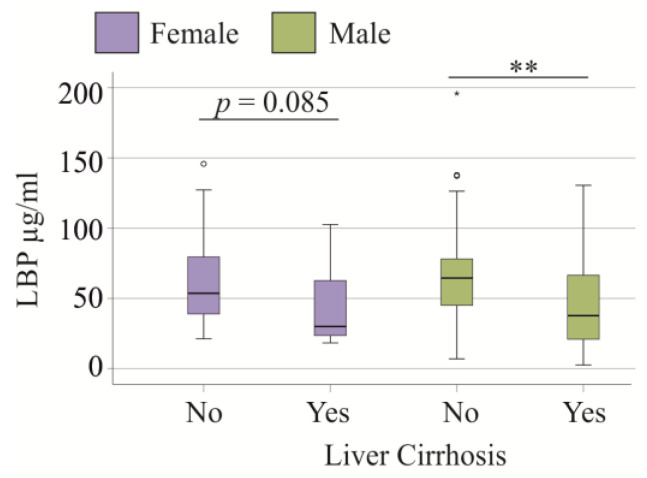
LBP in plasma of female and male SIRS/sepsis patients without (No) and with (Yes) liver cirrhosis. Statistical test used: Kruskall–Wallis test ** *p* < 0.01.

LBP is an acute-phase protein synthesized by hepatocytes and its circulating levels are increased in patients with liver cirrhosis compared to healthy controls [9,36]. However, in the SIRS/sepsis patient cohort, the 34 patients with liver cirrhosis had reduced plasma LBP levels (*p* = 0.001). The LBP of patients with cirrhosis was 32.4 (2.5–130.5) µg/mL and that of patients without cirrhosis was 52.2 (6.5–196.0) µg/mL. Among male patients, 26 had liver cirrhosis, and they also showed a decline in LBP levels in comparison to male patients with normal liver function (*p* = 0.002, Figure 2). The 8 females with liver cirrhosis had a trend for reduced LBP levels in comparison to female patients with normal liver function (*p* = 0.085) (Figure 2). Comparison of male with female patients without liver cirrhosis (*p* = 0.373) and male with female patients with liver cirrhosis (*p* = 0.687) revealed similar levels of plasma LBP (Figure 2).

In comparison to controls, patients with SIRS/sepsis and liver cirrhosis had higher LBP levels (*p* < 0.001 for both sexes). These data show that the plasma levels of LBP are increased in SIRS/sepsis patients with liver cirrhosis of both sexes but this increase is smaller in comparison to non-cirrhotic SIRS/sepsis patients.

Because of the low plasma LBP levels of patients with liver cirrhosis, these patients were not included in the further analyses.

### 3.3. Plasma LBP of SIRS/Sepsis Patients Stratified for SIRS, Sepsis, and Septic Shock and Underlying Diseases

**Figure 3:**
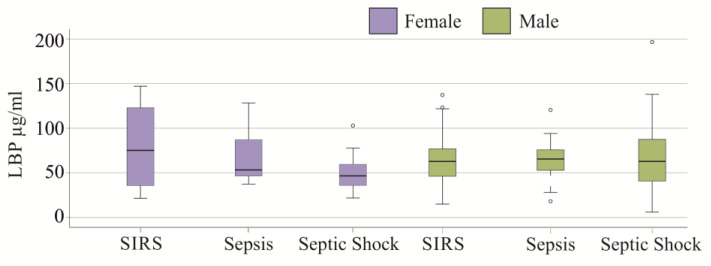
Plasma LBP levels of SIRS/sepsis patients without liver cirrhosis are categorized according to the SIRS criteria and the Sepsis-3 definition. Statistical test used: Kruskall–Wallis test.

Patients were divided into three cohorts: SIRS, sepsis, and septic shock [34]. There was no significant difference in circulating LBP levels between these groups, whether considered as an entire cohort (*p* = 0.375), by men (*p* = 0.990), or women (*p* = 0.082) (Figure 3).

Of our cohort, 41 patients developed SIRS/sepsis due to pancreatitis, and 9 due to cholangitis. Patients with pancreatitis and cholangiosepsis exhibited similar plasma LBP levels compared to all other patients (*p* = 0.820). Pancreatitis and cholangitis were diagnosed in four women each, with no significant difference in LBP levels compared to females without these underlying diseases (*p* = 0.842). Among men, the 37 patients with pancreatitis and the 5 patients with cholangiosepsis displayed LBP levels comparable to patients without these conditions (*p* = 0.816).

### 3.4. Plasma LBP of SIRS/Sepsis Patients Stratified for Infectious Diseases, SARS-CoV-2 and Bacterial Infections

Common infections that lead to sepsis were pulmonary (45 patients) and urinary tract infections (16 patients). Plasma LBP levels were similar between patients with pulmonary and urinary tract infections in comparison to patients with other causes of disease in the entire cohort (*p* = 0.828), in males (*p* = 0.707) and females (*p* = 0.266). The 9 females with urosepsis tended to have higher LBP levels in comparison to the female patients without urinary tract infections (*p* = 0.085). Plasma LBP levels of male and female patients with urosepsis were similar (*p* = 0.210).

Severe COVID-19 disease is a recent cause of sepsis [37,38]. However, there were no significant differences in LBP levels between the 18 male COVID-19 patients and non-infected males (*p* = 0.238), the 7 SARS-CoV-2 infected females and the non-infected females (*p* = 0.224) or in the entire cohort (*p* = 0.182).

### 3.5. Plasma LBP Levels in Relation to Vasopressor Therapy and Therapeutic Interventions

The associations of plasma LBP levels with the need for dialysis (12 females and 41 males), mechanical ventilation (27 females and 67 males), or vasopressor treatment (24 females and 76 males) were calculated for the SIRS/sepsis patients. Plasma LBP levels of patients requiring these interventions and those of patients without the need for these therapies were compared. Plasma LBP levels were not associated with any of these measures in the entire cohort or in the sex-specific analysis (*p* > 0.05 for all).

### 3.6. Plasma LBP Levels in Relation to Inflammation Markers

In the entire cohort, among male and female patients, a positive correlation was identified between plasma LBP and CRP as well as procalcitonin levels (Table 2). Positive correlations of LBP and IL-6 were significant in the whole cohort and in males. A positive correlation of LBP with basophils was detected in males and a negative with immature granulocytes in females (Table 2). It should be noted that there was no difference in procalcitonin, CRP, and interleukin-6 as well as immune cell counts between males and females (*p* > 0.05 for all).

### 3.7. Plasma LBP Levels in Relation to Markers of Liver Disease

In the SIRS/sepsis cohort plasma LBP negatively correlated with albumin (r = −0.254, *p* = 0.002) and positively with bilirubin (r = 0.268, *p* = 0.001). In the male SIRS/sepsis patients, plasma LBP positively correlated with alanine aminotransferase (r = 0.281, *p* = 0.004), aspartate aminotransferase (r = 0.224, *p* = 0.024), and bilirubin (r = 0.338, *p* < 0.001). In female SIRS/sepsis patients, the correlation with albumin was significant (r = −0.369, *p* = 0.017).

In SIRS/sepsis patients with liver cirrhosis, plasma LBP positively correlated with alanine aminotransferase (r = 0.442, *p* = 0.010), aspartate aminotransferase (r = 0.520, *p* = 0.002), and gamma-glutamyltransferase (r = 0.527, *p* = 0.003). In patients with liver cirrhosis, plasma LBP was also positively correlated with procalcitonin (r = 0.480, *p* = 0.004) and CRP (r = 0.703, *p* < 0.001) but not with IL-6 and immune cell numbers.

### 3.8. Plasma LBP Levels in Gram-Negative and Gram-Positive Infection

**Figure 4:**
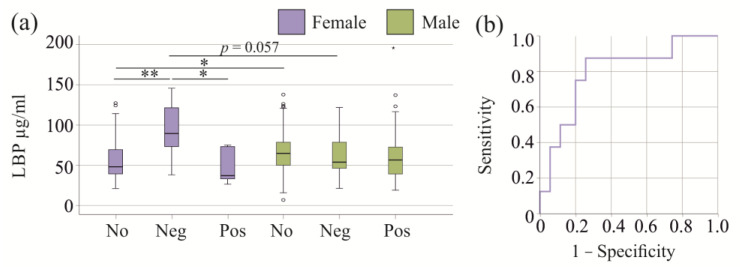
LBP in plasma of patients with SIRS/sepsis stratified for type of bacterial infection. (**a**) LBP in plasma of female and male patients with SIRS/sepsis stratified for type of bacterial infection; Statistical test used: Kruskal–Wallis test; (**b**) Receiver operating characteristic for discrimination of females with Gram-negative infections from those with no or Gram-positive infections by plasma LBP. * *p* < 0.05, ** *p* < 0.01.

Plasma LBP levels of the 19 patients with Gram-negative, the 21 patients with Gram-positive bacteria, and the 4 patients with both types of bacteria in their blood cultures were similar to those with negative blood cultures in the entire cohort (*p* = 0.148). Only one female SIRS/sepsis patient was infected with both types of bacteria and for sex-specific analysis patients infected with Gram-negative as well as Gram-positive bacteria were excluded. The 8 females with Gram-negative infection had higher plasma LBP levels in comparison to those with negative blood cultures (*p* = 0.004) and to those 5 patients with Gram-positive infections (*p* = 0.015; Figure 4a). The difference between male and female patients with Gram-negative blood cultures was almost significant (*p* = 0.057). Receiver operating characteristic curve analysis revealed an area under the curve of 0.796 (*p* = 0.010) for discrimination of females with Gram-negative bacteria from females with no/Gram-positive infections (Figure 4b). Plasma levels of 70 µg/mL LBP had a sensitivity of 88% and a specificity of 74% to detect Gram-negative infection in females.

Age (*p* = 0.937) did not significantly differ between non-infected and infected patients. Procalcitonin levels of females with Gram-negative infections were higher in comparison to non-infected (*p* = 0.007) and Gram-positive infected females (*p* = 0.019), whereas IL-6 and CRP levels were not changed.

Females without bacterial infections had lower plasma LBP levels than males without bacterial infections (*p* = 0.040, Figure 4a). CRP (*p* = 0.327) and procalcitonin (*p* = 0.646) did not differ between these groups.

### 3.9. Plasma LBP Levels and Survival

**Figure 5:**
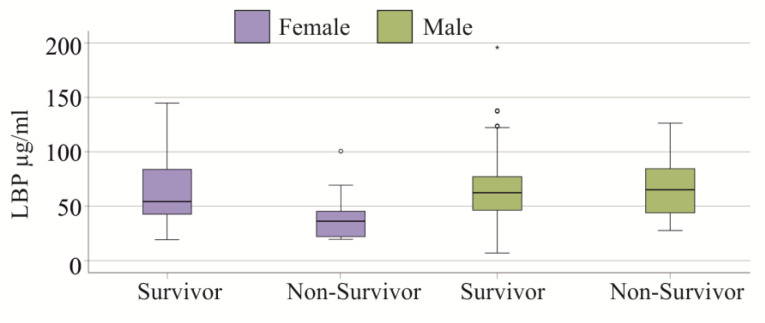
Association of plasma LBP levels with survival. Plasma LBP levels of the female and male SIRS/sepsis patients who survived and the patients who did not survive.

Plasma LBP levels of the 29 patients (patients with liver cirrhosis were excluded) who did not survive were similar to those patients who survived (*p* = 0.464). This was also observed for the 20 male non-survivors (*p* = 0.500, Figure 5). The 9 females who did not survive had reduced plasma LBP levels in comparison to women who survived (*p* = 0.017, Figure 5). Female non-survivors had lower LBP levels in comparison to male non-survivors (*p* = 0.011). These differences were only significant when pairwise comparisons were performed. Statistical analysis of the entire cohort did not reveal a significant difference (*p* = 0.083).

Immune cell counts, CRP, procalcitonin, age, and BMI of non-surviving males and females were similar (*p* > 0.05 for all).

## 4. Discussion

This is to our knowledge the first sex-specific analysis of plasma LBP in patients with SIRS/sepsis. This study shows for the first time that (1) female patients with Gram-negative bacterial bloodstream infections have higher plasma LBP levels than males. (2) Plasma LBP decreases in women who do not survive. (3) Sepsis-induced increases in LBP are impaired in cirrhosis.

Patients’ plasma was collected within 12 to 24 h of admission to intensive care to determine whether LBP is an early marker of disease severity, bacterial infection, and outcome. Early biomarkers of bacterial infection may serve as a diagnostic tool to help guide antibiotic treatment decisions [39,40]. It has been shown that serum levels of LBP in patients with sepsis decrease modestly over 4 to 5 days of follow-up [5,12]. A decrease in serum LBP levels was also observed at 48 h, with a further decrease on day 7 [41]. LBP levels in survivors showed a more pronounced decrease compared to non-survivors in one of these studies [41] which was not observed in others [10,12]. Further studies are therefore needed to show whether monitoring LBP during follow-up is of diagnostic value.

LBP levels were significantly elevated in patients with SIRS/sepsis in comparison to healthy controls, as demonstrated by several studies [10,11,12,42]. Men have a higher prevalence of sepsis [19], but sex-specific analysis of LBP levels in patients with SIRS/sepsis is lacking. The current data showed a similar increase in LBP in sepsis in both male and female patients. Plasma LBP levels of males and females with the need for interventions or vasopressor therapy were similar. Moreover, patients with SIRS, sepsis, and septic shock had comparable plasma LBP levels in both sexes. This is in accordance with a meta-analysis showing that LBP is not a diagnostic marker for the discrimination of SIRS and sepsis [42].

Whether LBP can discriminate between infectious and non-infectious severe illness remains to be determined [10,11,12,42]. The current analysis showed that women with Gram-negative infections had increased plasma LBP levels compared with women with negative blood cultures and women with Gram-positive infections. Although this group of patients was small, the difference was significant, such that larger studies need to be performed to test the ability of LBP to early diagnose Gram-negative bloodstream infections in females. The median age of the women was 60 years and did not differ between uninfected, Gram-negative infections, and Gram-positive infections. In addition, the CRP levels of these women were similar. Women with Gram-negative infections had higher procalcitonin levels and procalcitonin has been identified as a marker helpful in recognizing infections with Gram-negative bacteria. However, sex-specific studies are still missing to our knowledge [43]. The mechanisms associated with higher plasma LBP levels in women infected with Gram-negative bacteria need to be investigated further.

In cases of urosepsis, females tended to have increased plasma LBP levels, while this trend was not observed in males. It is worth noting that urosepsis is mainly caused by Gram-negative bacteria [44], and in our study, four out of nine women with urosepsis had Gram-negative bacteria in their blood, which may contribute to modestly increased LBP levels. In female patients with pneumonia, only two had Gram-negative bacteria in their blood and LBP levels of these patients were similar to those without pneumonia.

Females generally have stronger innate and adaptive immune responses than males. These stronger immune responses enable better clearance of pathogens but also increase females’ susceptibility to inflammatory and autoimmune diseases [45]. The expression of the pattern recognition receptors TLR7 and activation of macrophages by TLR4 ligands are higher in females than males [46]. Additionally, women were also found to have a stronger inflammatory response to endotoxin injections [22]. It is worth noting that LBP is essential for TLR4 activation by LPS [36] and the sex disparity of LBP in Gram-negative infection—shown in our analyses—may contribute to the stronger immune response of females. In contrast to Gram-negative infected SIRS/sepsis patients in our study, male mice exhibited elevated LBP levels when challenged with LPS in comparison to female animals [20]. It should be noted that a higher inflammatory response in female mice was reported in another study using much older animals [21]. The immune response of both sexes changes with age, which is also related to the reduced protective effects of estrogen in females [24,25].

In fact, LBP has been shown to play a dual role in inflammation. LPS-induced activation is enhanced by low levels of LBP, whereas high levels of LBP inhibit inflammation [6,7,8].

In SIRS/sepsis patients without bacterial infection, women had lower plasma LBP levels than men, whereas CRP and procalcitonin did not differ between these groups. This difference was modest and is unlikely to be of pathophysiological relevance.

Patients with sepsis and liver cirrhosis have lower LBP and CRP levels compared to sepsis patients without liver cirrhosis [47]. Regarding LBP, previous studies showed higher LBP in patients with liver cirrhosis whose levels were further induced by infection [9,28]. Albillos et al. observed normal plasma LBP levels in patients with liver cirrhosis which were increased in liver cirrhosis patients with ascites [48]. In patients with non-alcoholic fatty liver disease, a decrease in LBP was observed with increasing severity of liver disease [49]. To our knowledge, no study has been published comparing LBP in SIRS/sepsis patients with and without cirrhosis. High levels of LBP were associated with a lower neutrophil respiratory burst in cirrhosis, and further studies are needed to clarify the role of LBP in patients with SIRS/sepsis and liver cirrhosis, which is associated with more severe sepsis and worse outcome [30].

In the overall SIRS/sepsis cohort and in patients with liver cirrhosis, plasma LBP levels were positively correlated with markers of liver disease severity. This is in line with a previous study showing positive correlations of LBP with aspartate aminotransferase and gamma-glutamyltransferase and a negative correlation with albumin in patients with liver cirrhosis [50]. In cirrhotic patients with sepsis, serum LBP was negatively correlated with the model for end-stage liver disease score and the Child-Pugh score and decreased in patients with severe liver dysfunction [51]. LBP in blood is therefore positively associated with markers of liver disease severity and decreases when hepatic synthesis is severely impaired. An impaired CRP response to *E. coli* bacteremia in patients with liver dysfunction has been shown before [27,47]. In patients with cirrhosis, plasma LBP correlated positively with CRP and procalcitonin. This shows that LBP levels are still related to inflammation in patients with SIRS/sepsis and cirrhosis, but the induction seems to be impaired, probably because of reduced hepatic synthesis.

In the entire cohort, as well as in male and female patients, plasma LBP showed a positive correlation with CRP and procalcitonin. Positive associations with interleukin-6 were observed in males, but not in females, which may be due to the smaller number of women with SIRS/sepsis. In female patients, plasma LBP was negatively correlated with immature granulocyte numbers. In male plasma, LBP was positively related to basophils. Immature granulocyte number is an early marker of sepsis and discriminates infected and non-infected patients but is not correlated with mortality in severe illness [52,53]. Reduced number of basophils is related to mortality in sepsis patients [54]. Currently, the sex-specific associations of plasma LBP levels with these immune cells are unclear and need further study.

In severe cases, SARS-CoV-2 infection is associated with elevated LBP, as reported by Sun et al. in 2022 [15]. Our study found that sepsis patients, regardless of COVID-19 status, had similar plasma LBP levels in both sexes. This suggests that higher levels of LBP in COVID-19 patients are related to critical illness and are not specifically induced by viral infection.

Our observation in the entire SIRS/sepsis cohort is consistent with the finding that systemic LBP measured early after admission to intensive care is not related to mortality [11,41,55,56]. Of clinical relevance, our sex-specific analyses revealed that female patients who did not survive had lower plasma LBP levels compared to both surviving females and compared to male patients who did not survive. We therefore suggest that this reduction of LBP in critically ill female patients is related to mortality. Immune cell counts, CRP and procalcitonin of male and female non-survivors were similar, and differences in inflammatory responses do not seem to explain this difference. It has been observed that lower LBP levels are associated with survival. This cohort consisted of 59% males and approximately 10% of the patients had hepatic dysfunction. However, a sex-specific analysis has not been conducted [57].

Age and BMI may be confounding factors in observational studies. However, in this study, there was no correlation between age and BMI and plasma LBP levels in both females and males. Serum LBP levels correlate positively with metabolic syndrome [58], atherosclerosis [59], and type 2 diabetes [60]. LBP may serve as a marker for “systemic chronic low-grade inflammation”, a state that characterizes a number of common diseases [61]. Interventions to lower systemic inflammation may also result in reduced serum LBP levels. Weight loss but not metformin treatments reduced serum LBP levels by 1 µg/mL within 1 year despite similar weight loss in both cohorts. This latter cohort did not show a decline in high-sensitive CRP in line with a positive association of serum LBP levels with inflammation [62]. Patients with and without coronary artery disease had median serum LBP levels of 6.8 and 6.1 µg/mL, respectively [63]. A second study reported mean LBP concentrations of 20.6 and 17.1 pg/mL for patients with and without angiographically confirmed coronary artery disease [64]. This shows that the effect of metabolic disease on serum LBP levels is small compared to its increase in SIRS/sepsis.

Plasma LBP levels of male and female SIRS/sepsis patients were similar. Plasma LBP levels of healthy controls did not differ by sex, consistent with literature data [65]. We did not document the BMI of our control cohort and this is a limitation of our study. The small number of females with bacterial infection is also a limitation of this analysis and the suitability of plasma LBP as an early marker for Gram-negative infections needs validation in larger cohorts. The number of female patients who did not survive was also limited and future study has to evaluate whether low plasma LBP is related to survival in female patients with SIRS/sepsis. Common comorbidities such as diabetes or metabolic-associated fatty liver disease were not documented.

## 5. Conclusions

The current study analyzed in detail the sex-specific associations of plasma LBP in a cohort of septic patients. Our analysis indicated that higher LBP plasma levels could serve as a valuable biomarker for Gram-negative bloodstream infections in females, while lower levels are related to mortality in this group. If our findings are confirmed in larger multicentric studies, these results could have a significant clinical impact by enabling early targeted antibiotic therapy in female septic patients.

## Figures and Tables

**Table 1:** Characteristics of controls, SIRS/sepsis patients, and SIRS/sepsis patients where patients with liver cirrhosis were excluded. Numbers in superscript refer to patients for whom these data were available when data were not collected for the entire cohort. Statistical tests used: Mann–Whitney U-test (for age), Wilcoxon test (for all other parameters listed in Table 1), and Chi-square test (for sex distribution) * *p* < 0.05, *** *p* < 0.001.

Parameters	All Patients	Patients with Liver Cirrhosis Excluded	Controls
Males/Females	138/51	112/43	25/18
Age (years)	60 (21–93) *	60 (21–93) *	56 (21–86) *
Body Mass Index (kg/m^2^)	27.0 (15.4–55.6) ^186^	27.0 (15.4–55.6) ^152^	not defined
SIRS/Sepsis/Septic Shock	46/51/92	40/40/75	not defined
C-reactive protein mg/L	154 (4–697) ***	177 (3.6–697) ***	not defined
Procalcitonin ng/mL	1.31 (0.05–270.00) ^186^	1.48 (0.05–270.00) ^152^	not defined
Interleukin-6 pg/mL	98 (0–107,039) ^167^	90 (0–107,039) ^135^	not defined
Leukocytes n/nL	10.47 (0.06–1586.00)	10.47 (0.06–246.94)	not defined
Neutrophils n/nL	7.98 (0–70.20) ^183^	7.98 (0–70.20) ^149^	not defined
Basophils n/nL	0.04 (0–0.90) ^184^	0.04 (0–0.90) ^150^	not defined
Eosinophils n/nL	0.10 (0–8.80) ^184^	0.09 (0–8.80) ^150^	not defined
Monocytes n/nL	0.78 (0–45.00) ^184^	0.76 (0–45.00) ^150^	not defined
Lymphocytes n/nL	0.93 (0.08–28.60) ^184^	0.97 (0.08–28.60) ^150^	not defined
Immature Granulocytes n/nL	0.14 (0–7.25) ^182^	0.16 (0–7.25) ^148^	not defined
Aspartate aminotransferase U/L	48 (6–8084) ^176^	44 (6–8084) ^143^	not defined
Alanine aminotransferase U/L	32 (5–1042) ^173^	32 (5–1042) ^140^	not defined
Albumin g/L	23.8 (6.3–42.0) ^177^	24.1 (6.3–42.0) ^144^	not defined
Gamma-glutamyltransferase U/L	127 (11–1266) ^158^	131 (11–1266) ^129^	not defined
Bilirubin mg/dL	1.00 (0.10–36.60) ^181^	0.80 (0.10–23.90) ^147^	not defined

**Table 2:** Correlation coefficients (r) for plasma LBP levels and their associations with clinical markers of inflammation. Statistical test used: Spearman correlation.

Biomarker of Inflammation	Entire Cohort Without Liver Cirrhosis	Females Without Liver Cirrhosis	Males Without Liver Cirrhosis
	r	r	r
Procalcitonin	0.286 ***	0.412 **	0.225 *
C-reactive protein	0.392 ***	0.358 *	0.416 ***
Interleukin-6	0.318 ***	0.266	0.347 **
Leukocytes	−0.044	−0.172	0.017
Neutrophils	0.020	−0.253	0.145
Basophils	0.113	−0.081	0.202 *
Eosinophils	0.032	0.066	0.050
Monocytes	0.023	−0.123	0.085
Lymphocytes	−0.031	−0.134	0.024
Immature Granulocytes	0.028	−0.331 *	0.146

* *p* < 0.05, ** *p* < 0.01, *** *p* < 0.001.

## Data Availability

Data supporting reported results can be obtained from the corresponding author.
